# Evaluating the educational effectiveness of team-based cadaveric dissection in undergraduate anatomy training

**DOI:** 10.1186/s12909-026-09491-2

**Published:** 2026-06-09

**Authors:** Willy Barinem Vidona, Helen Kikelomo Akinsunmi, Ikenna Theophilus Ikele

**Affiliations:** 1 Department of Anatomy, Ambrose Alli University, Ekpoma, Edo State Nigeria; 2 Department of Anatomy, Turnbull Centre of Basic Sciences, Queen Mary University of London, London, UK

**Keywords:** Anatomy education, Cadaveric dissection, Team-based learning, Collaborative learning, Medical students, Resource-limited settings

## Abstract

**Background:**

Cadaveric dissection remains central to undergraduate anatomy education, yet its effectiveness is increasingly constrained by limited cadaver availability, inadequate laboratory infrastructure, and the emotional burden experienced by students. These challenges highlight the need for pedagogical approaches that optimise learning while maximising scarce anatomical resources. This study evaluated the educational effectiveness of a team-based learning (TBL) approach to cadaveric dissection among undergraduate anatomy students at Ambrose Alli University, Ekpoma, Nigeria.

**Methods:**

A quasi-experimental design was employed involving 155 second-year anatomy students assigned to either a traditional learning (TL) group (*n* = 77) or a TBL group (*n* = 78). Academic performance was assessed using standardised examination scores. Students’ perceptions of collaboration, motivation, critical thinking, emotional adaptation, and resource utilisation were collected using structured questionnaires. Data were analysed using descriptive statistics and independent-samples t-tests, with statistical significance set at *p* < 0.05.

**Results:**

Students in the TBL group achieved significantly higher mean examination scores (84.6 ± 6.8) than those in the TL group (74.2 ± 7.5; t = 8.12, *p* < 0.001). A greater proportion of TBL participants reported enhanced collaboration (88% vs. 52%), increased motivation (90% vs. 60%), and improved emotional adjustment to dissection activities (82% vs. 48%). Additionally, 85% of TBL students indicated that task distribution within teams improved cadaver utilisation, compared with 38% in the TL group.

**Conclusions:**

Team-based cadaveric dissection produced superior cognitive and affective learning outcomes compared with traditional instruction. By improving academic performance, fostering teamwork, reducing emotional stress, and enhancing the efficient use of limited cadaveric resources, TBL represents a practical and effective pedagogical model for anatomy education in resource-constrained settings.

**Supplementary Information:**

The online version contains supplementary material available at 10.1186/s12909-026-09491-2.

## Introduction

Anatomy remains a foundational component of medical and health-professions education, underpinning essential clinical competencies such as diagnosis, surgical practice, and radiological interpretation [1]. Despite the increasing availability of digital and virtual learning tools, cadaveric dissection remains the most authentic and immersive method for learning human anatomy, consistently enhancing spatial understanding and critical thinking among undergraduate learners [2, 3]. In Nigeria, this preference persists, with most students reporting that dissection deepens comprehension and supports cognitive development [4].

However, the sustainability of cadaver-based anatomy education is increasingly challenged. Expanding student enrolments, compressed curricular schedules, and limited laboratory infrastructure place significant pressure on existing teaching resources [5]. In Nigeria, these challenges are compounded by persistent shortages of cadavers. Most institutions rely on unclaimed bodies due to the absence of a centralised body donation system, and cultural and religious beliefs further limit voluntary donation [6, 7]. Even among anatomists, willingness to donate remains low, contributing to cadaver-to-student ratios that can exceed 20:1 [6].

Traditional dissection in Nigeria typically follows a teacher-centred model, with students working individually or in loosely structured groups [4]. In recent years, however, anatomy educators have advocated for more interactive and student-centred pedagogies, including problem-based learning, blended digital approaches, and team-based learning (TBL). TBL is a structured, collaborative instructional strategy that emphasises accountability, peer interaction, and application-focused learning. Evidence from health-professions education shows that TBL improves academic performance, strengthens teamwork and communication skills, and enhances engagement with complex anatomical concepts [8–10]. When applied in cadaver laboratories, TBL has been shown to increase the educational value of limited cadaveric resources and promote deeper learning [9].

Despite these promising findings, most existing research on TBL in cadaveric anatomy laboratories originates from Europe, North America, and Asia. Empirical evidence from African contexts, particularly Nigeria, remains scarce [11]. This gap is significant given the unique challenges faced by Nigerian anatomy programmes, including cadaver scarcity, infrastructural limitations, and the emotional stress associated with dissection. Students frequently report anxiety, discomfort, and psychological strain during early exposure to cadavers, and structured peer-supported approaches such as TBL may help mitigate these emotional burdens while enhancing learning outcomes [12].

Recent systematic reviews highlight the effectiveness of team-based learning in health professions education [13] A meta-analysis reported that TBL significantly improves knowledge acquisition compared to lecture-based teaching (standardised mean difference = 0.68, 95% CI: 0.45 to 0.91) [14].

These contextual realities underscore the need to evaluate the pedagogical value of TBL within Nigerian anatomy education. Understanding how TBL influences academic performance, teamwork, motivation, and emotional adjustment can inform evidence-based curriculum reforms. Additionally, examining how TBL affects cadaver utilisation and laboratory efficiency is essential for resource-limited settings where maximising educational yield is a priority.

This study, therefore, investigates the educational effectiveness of team-based cadaveric dissection among undergraduate anatomy students at Ambrose Alli University, Ekpoma. Specifically, it examines the impact of TBL on academic achievement, collaborative and critical-thinking skills, student motivation, emotional adaptation to dissection, and the utilisation of limited cadaveric resources. The findings aim to contribute to the growing literature on anatomy pedagogy and provide practical recommendations for enhancing anatomy training in resource-constrained environments.

## Materials and methods

### Study design

A quasi-experimental, descriptive study was conducted to evaluate the educational effectiveness of team-based learning (TBL) compared with traditional learning (TL) during cadaveric dissection sessions. The study was carried out in the Department of Anatomy, Ambrose Alli University, Ekpoma, Edo State, Nigeria.

### Setting

Ambrose Alli University is a state-owned institution with a student population exceeding 10,000, including large cohorts in medicine, health sciences, and basic medical sciences. Gross anatomy practical sessions are conducted in the departmental dissection laboratory, where cadaveric dissection forms a core component of the curriculum.

### Participants

The target population comprised undergraduate anatomy students aged 18–45 years who were actively participating in gross anatomy dissection. Only second-year students enrolled in the anatomy programme during the study period were eligible. A total of 155 students participated.

### Sample size determination

The minimum sample size (*n* = 155) was calculated using the Taro Yamane formula (1976):

$$[ n = \frac{N}{1 + N(e)^2} ]$$

where *N* is the population size and *e* is the margin of error (0.05). The calculation was based on the estimated undergraduate population within the Faculty of Basic Medical Sciences.

### Sampling technique

Purposive sampling was used to select students with direct experience in cadaveric dissection. This ensured that participants possessed relevant exposure to the instructional methods being evaluated, thereby enhancing the validity of the findings.

### Inclusion criteria

Participants were eligible if they:

- were aged 18 years or older
- were enrolled as second-year anatomy students
- were present within the university environment during the study period
- provided informed consent and completed the questionnaire

### Data collection methods

#### Group allocation procedure

Participants were allocated to either the Traditional Learning (TL) group or the Team-Based Learning (TBL) group using a non-randomised, quasi-experimental design based on existing class sections. The study was conducted during the 2024/2025 academic session. Two parallel streams of second-year anatomy students were enrolled in the gross anatomy course. Stream A (*n* = 77) was assigned to the TL group, and Stream B (*n* = 78) was assigned to the TBL group. Assignment was determined by the university’s pre-existing course registration schedule, not by the research team. Both streams followed the same curriculum, had the same instructor-to-student ratio (1:15), used the same cadaveric specimens, and covered identical anatomical regions over the same 8-week period.

### Instructional interventions

#### Traditional Learning (TL) group

Students in the TL group received teacher-centred, lecture-based instruction. The instructor guided students through dissection procedures, highlighted key anatomical structures, and summarised essential concepts. Students were required to pre-read assigned textbook chapters (e.g., *Atlas of Anatomy*, *Cunningham’s Manual of Practical Anatomy*) and could ask questions during or after practical sessions. Each student completed pre-- and post-session tests to assess learning outcomes.

#### Team-Based Learning (TBL) group

Students assigned to the TBL group received structured collaborative instruction. One week before the session, students received preparatory materials and handouts. Groups of 4–5 students (balanced by gender and characteristics) completed individual readiness tasks, followed by group discussions to generate consensus answers. During dissection, groups worked collaboratively on cadavers and anatomical models. Peer-evaluation forms were completed at the end of each session. Pre- and post-tests were administered to assess learning gains.

The TBL method included an “Individual Readiness Assurance Task” (iRAT), which consisted of 10 multiple-choice questions based on the pre-reading assignment. Each student completed the iRAT independently and without consultation within the first 10 min of the laboratory session. The iRAT assessed basic anatomical knowledge (e.g., the identification of muscles, nerves, and vessels) and helped ensure individual accountability before group work began. Following the iRAT, students completed the same questions again as a “Group Readiness Assurance Task” (gRAT) through team discussion, providing one consensus answer per group.

Both instructional models were adapted from previously validated approaches in anatomy education [15].

This study implemented a modified version of the established Michaelsen model of Team-Based Learning [16]. The TBL intervention consisted of the following core components delivered over each of the 8 laboratory sessions:

#### Phase 1: pre-class preparation (1 week prior)

One week before each laboratory session, students received a detailed handout outlining the learning objectives, pre-reading assignments (from Cunningham’s Manual of Practical Anatomy and Atlas of Anatomy), and a set of 10 preparatory multiple-choice questions. Students were required to complete the pre-reading independently before the session.

#### Phase 2: readiness assurance process (first 30 min of each 3-hour session)

##### Individual readiness assurance test (iRAT)

Each student completed a 10-question multiple-choice test independently, without consultation, based solely on the pre-reading. The iRAT accounted for 10% of the session grade.

##### Group readiness assurance test (gRAT)

Immediately following the iRAT, the same 10 questions were completed by each team (4–5 students) through consensus discussion. Teams selected a single answer sheet to submit, and the instructor provided immediate feedback. The gRAT accounted for 10% of the session grade.

##### Appeals process

Teams were permitted to submit written appeals for any gRAT question they believed had more than one correct answer or was ambiguously worded. Appeals were reviewed by the instructor before the next session.

#### Phase 3: application exercise (60–90 min during dissection)

Following readiness assurance, teams engaged in structured application exercises while dissecting cadavers. Each exercise presented a clinically relevant problem (e.g., “A patient presents with inability to abduct the shoulder. Identify the nerve involved and trace its course on the cadaver.“). Teams were required to: (1) discuss the problem as a group; (2) locate relevant anatomical structures on the cadaver; (3) formulate a consensus answer; and (4) report their answer simultaneously using colour-coded cards (a “4S” framework: same problem, same choice, simultaneous report). The instructor facilitated debriefing after each application exercise.

#### Phase 4: peer evaluation (last 10 min of selected sessions)

At the end of sessions 2, 4, 6, and 8, students completed anonymous peer evaluation forms assessing each team member’s contribution, preparation, and respect for others. Peer evaluation scores accounted for 5% of the final grade.

##### Facilitation

Each TBL session was facilitated by two anatomy lecturers with skills in TBL facilitation. Facilitators circulated among teams, answered clarifying questions, and led whole-class debriefings. Facilitators did not provide direct answers during the gRAT or application exercise phases.

**Peer Evaluation and Group Composition**

The TL group consisted of all 77 students who participated in the traditional dissection sessions. Within the TL group, students were not formally divided into fixed small subgroups for peer evaluation purposes. Instead, students worked in an open laboratory setting where they could interact voluntarily with teachers and peers. While students in the TL group naturally formed informal clusters during dissection, no structured peer evaluation was administered to them, as this is not part of the traditional teaching method at this institution.

### Laboratory sessions and anatomical regions

The study was conducted over 8 laboratory sessions, each lasting 3 h, held weekly for 8 weeks. The anatomical regions covered during these sessions were: (1) upper limb (shoulder, arm, forearm, and hand); (2) lower limb (gluteal region, thigh, leg, and foot); (3) thorax (heart, lungs, and mediastinum); and (4) abdomen (gastrointestinal tract, liver, and kidneys). Each region was covered over two consecutive laboratory sessions (6 h total per region). The same anatomical regions were covered in both the TL and TBL groups, and the same cadaveric specimens were used across both groups.

### Data collection instruments

#### Academic performance test

A standardised examination (maximum score: 100) was administered after the TL and TBL sessions. Scores were categorised as:

- Excellent: 90–100
- Good: 80–89
- Moderate: 70–79
- Pass: 60–69

The number of students in each performance category was recorded for both groups.

##### Test score assessment

Both TL and TBL groups completed a pre-test (week 0) and a post-test (week 9). The pre-test consisted of 30 multiple-choice questions covering general anatomical knowledge of the four regions assessed. The post-test consisted of 50 multiple-choice questions (30 identical to the pre-test plus 20 new questions assessing deeper application) and 2 short-answer questions. Pre-test and post-test scores are reported as mean percentage ± standard deviation. Learning gain was calculated as (post-test % – pre-test %).

#### Structured questionnaire

A validated questionnaire assessed students’ perceptions of:

- communication ability
- expression and explanation skills
- generalisation and conceptualisation
- collaboration
- knowledge extension
- learning initiative
- classroom atmosphere

Items were rated on a 3-point Likert scale (“agree,” “disagree,” “neither”). Questionnaire validity was established through expert review by three anatomy lecturers. All 155 questionnaires were completed and returned (100% response rate).

A 3-point Likert scale was chosen instead of a 5-point scale for the following reasons: (1) to minimize central tendency bias, where respondents may just select neutral midpoints (e.g., “neither agree nor disagree” on a 5-point scale); (2) to ensure clarity and ease of interpretation for the student population, many of whom are non-native English speakers; and (3) previous validation studies in similar Nigerian educational contexts have demonstrated that 3-point scales yield comparable reliability coefficients (Cronbach’s α > 0.80) while reducing ambiguous responses [17].

### Reliability of the questionnaire

Face and content validity of the questionnaire were established through review by three experienced anatomy lecturers from the Department of Anatomy, Ambrose Alli University, Ekpoma. Based on their feedback, minor revisions were made to item wording for clarity. To assess internal consistency reliability, the questionnaire was pilot-tested on 30 students (15 from TL, 15 from TBL) who were not part of the main study sample. The pilot data yielded a Cronbach’s alpha coefficient of 0.84, indicating good internal consistency. The reliability of the 3-point Likert scale in this context was further supported by item-total correlations ranging from 0.62 to 0.79.

### Clarification of comparability between TL and TBL methods

To ensure a fair comparison between the Traditional Learning (TL) and Team-Based Learning (TBL) methods, the following measures were implemented.

First, the preparatory materials were identical across both groups. Specifically, both TL and TBL students were assigned the same pre-readings from the same anatomy textbooks, covering the same anatomical regions (upper limb, lower limb, thorax, abdomen) for each corresponding session.

Second, both groups received their pre-reading assignments one week prior to the laboratory session to allow adequate preparation time.

Third, the same learning objectives and anatomical content were covered in both groups. The key difference between the two methods was the instructional format: TL was teacher-directed with individual or whole-class interaction, while TBL incorporated structured small-group discussion, peer accountability, and team-based problem-solving during dissection.

### Data analysis

All statistical analyses were performed using SPSS version 26.0. After being tested for homogeneity of variance, the examination scores of the students in the TL and TBL groups were analysed by using one-way analysis of variance with the Tukey–Kramer post hoc tests. Additionally, the effect of the 2 methods was evaluated using the χ2 test. For continuous variables (examination scores), the Shapiro-Wilk test indicated normal distribution (*p* > 0.05). Therefore, parametric tests were employed. To compare examination scores between the two independent groups (TL vs. TBL), an independent-samples t-test was used. Mean differences and 95% confidence intervals (CIs) are reported for all primary outcomes. For categorical variables (questionnaire responses), the chi-square (χ²) test was used to compare the proportion of students who “agreed” with each statement between the TL and TBL groups. For 2 × 2 tables with expected cell frequencies below 5, Fisher’s Exact Test was applied. A *p* < 0.05 was considered significant.

### Ethical considerations

Ethical approval was obtained from the Ambrose Alli University Research Ethics Committee (Approval No. 150/25). Participation was voluntary, and informed consent was obtained from all students. Hence, participation did not influence academic grading (no potential coercion). No personal identifiers (names, matriculation numbers) were collected to ensure confidentiality.

## Results

### Academic performance under traditional and team-based learning

A total of 155 students completed the study (TL: *n* = 77; TBL: *n* = 78). Student performance differed markedly between the two instructional approaches. As shown in Table 1, a greater proportion of students in the TBL group achieved excellent (22 students) and good grades (40 students), whereas the majority of students in the TL group scored within the moderate range (42 students). Only five students in the TL group achieved excellent scores compared with 22 in the TBL group.

**Table 1:** Distribution of student performance in TL and TBL groups

Grade Category	Traditional Learning (TL)	Team-Based Learning (TBL)
Excellent (90–100)	5 (6.5%)	22 (28.2%)
Good (80–89)	18 (23.4%)	40 (51.3%)
Moderate (70–79)	42 (54.5%)	18 (23.1%)
Pass (60–69)	20 (26.0%)	5 (6.4%)
Excellent (90–100)	5	22
Good (80–89)	18	40
Moderate (70–79)	42	18
Pass (60–69)	20	5

These findings indicate that TBL was associated with higher academic achievement and a shift toward higher-level performance categories

The mean examination score for the TBL group (84.6 ± 9.2) was significantly higher than that of the TL group (72.3 ± 10.5). The mean difference was 12.3 points (95% CI: 9.1 to 15.5). Independent samples t-test revealed a statistically significant difference between groups [t(153) = 7.82, *p* < 0.001].

### Development of collaborative and critical-thinking skills

Students in the TBL group reported greater improvements across all measured skill domains compared with those in the TL group (Table 2). Notably, collaboration (85%) and critical thinking (88%) were the most enhanced attributes among TBL participants, compared with 52% and 58%, respectively, in the TL group.

**Table 2:** Students’ perception of skills development in TL and TBL groups

Skill/Attribute	TL (*n* = 77)	TBL (*n* = 78)	χ²	*p*-value
Communication ability	55%	82%	12.84	< 0.001
Generalization ability	50%	80%	14.92	< 0.001
Collaboration ability	52%	85%	19.23	< 0.001
Critical thinking	58%	88%	17.45	< 0.001

These results suggest that TBL fostered more effective peer interaction, deeper reasoning, and improved communication during cadaveric dissection

### Motivation, emotional adjustment, and learning experience

Students exposed to TBL reported higher motivation, better emotional adjustment, and reduced stress during dissection activities (Table 3). Ninety per cent of TBL students indicated increased motivation compared with 60% in the TL group. Similarly, 82% of TBL students reported positive emotional adjustment, compared with 48% in the TL group.

**Table 3:** Students’ perceptions of motivation and emotional adjustment

Perception/Response	Traditional Learning (TL)	Team-Based Learning (TBL)
Increased motivation	60%	90%
Positive emotional adjustment	48%	82%
Stress reduction during dissection	40%	75%
Perceived interactive atmosphere	57%	87%

These findings indicate that TBL created a more supportive and interactive learning environment, reducing emotional strain associated with cadaveric dissection

### Cadaveric resource utilisation

Students in the TBL group perceived the use of cadaveric resources as more efficient than those in the TL group (Table 4). 85% of TBL students agreed that task-sharing improved cadaver utilisation, compared with 38% in the TL group. Additionally, fewer TBL students felt that limited cadaver numbers negatively affected learning (50% vs. 70%).

**Table 4:** Students’ opinions on cadaveric resource utilisation

Statement	Traditional Learning (TL)	Team-Based Learning (TBL)
Cadavers were adequately utilised	45%	80%
Task-sharing improved the use of cadavers	38%	85%
Limited cadaver numbers affected learning	70%	50%
Overall efficiency of cadaver usage	52%	88%

These results suggest that TBL enhanced the educational yield of limited cadaveric resources through structured teamwork and shared responsibilities

### Laboratory infrastructure and learning outcomes

Although laboratory infrastructure posed challenges for both groups, students in the TBL group rated its overall impact more positively (Table 5). Adequacy of space (72%) and availability of dissection tools (70%) were rated more favourably by TBL students, likely due to collaborative adaptation and shared task management.

**Table 5:** Students’ Ratings of Laboratory Infrastructure Influence

Laboratory Factor	Negative Impact (TL)	Positive Impact (TBL)
Lighting and ventilation	65%	55%
Availability of dissection tools	58%	70%
Adequacy of space	60%	72%
Overall impact on learning	50%	78%

These findings indicate that teamwork mitigated some infrastructural limitations, enabling more effective engagement with the dissection environment

## Discussion

This study compared outcomes between a traditional passive dissection method and a multi-component team-based learning intervention. The findings suggest associations rather than causal effects, and the specific drivers of observed differences cannot be determined from this design. Students in the TBL group achieved significantly higher test scores than those in the TL group, reinforcing evidence that TBL enhances academic performance and long-term retention of knowledge [18, 19]. While these findings align with previous research, it remains important to determine whether such gains are sustained over time and across diverse student populations, as many existing studies involve relatively homogenous cohorts. Factors such as group dynamics, instructor expertise, and prior exposure to collaborative learning may also influence the magnitude of improvement and warrant further investigation.

Similar improvements have been documented in pathology and biochemistry courses, where TBL significantly outperformed lecture-based instruction [20, 21]. However, it is unclear whether TBL is equally effective across all disciplines or whether its benefits are amplified in subjects requiring active engagement and problem-solving. Anatomy, with its reliance on spatial reasoning and hands-on practice, may uniquely favour TBL, but the transferability of these findings to more abstract subjects remains an open question.

There was no significant difference between groups on the pre-test [TL: 58.4% ± 7.2%; TBL: 59.1% ± 6.9%; t(153) = 0.62, *p* = 0.54, Cohen’s d = 0.10]. Following the intervention, the TBL group demonstrated significantly higher post-test scores [TL: 72.3% ± 10.5%; TBL: 84.6% ± 9.2%; t(153) = 7.82, *p* < 0.001, Cohen’s d = 1.26]. The mean learning gain (post-test minus pre-test) was 13.9% for the TL group and 25.5% for the TBL group (mean difference = 11.6%, 95% CI: 8.9% to 14.3%, *p* < 0.001). These findings indicate that the TBL group demonstrated greater performance gains over the course of the study.

The results also highlight the importance of active learning strategies in anatomy, where traditional didactic approaches have been criticised for promoting passive learning [2]. By engaging students collaboratively in cadaveric dissection, TBL appears to bridge gaps in comprehension, engagement, and retention. Nonetheless, it is worth considering whether improvements arise solely from the TBL framework or partly from increased accountability and peer influence inherent in group settings. Group dynamics may also introduce inequities, as dominant personalities can overshadow quieter members, potentially affecting individual learning outcomes.

TBL significantly enhanced communication, collaboration, and critical-thinking skills, with over 85% of participants reporting improvements. This aligns with earlier findings that TBL fosters teamwork, accountability, and higher-order reasoning in medical education [22, 23]. However, these outcomes were self-reported, raising the possibility of response bias. Future studies should incorporate validated assessment tools to objectively measure soft-skill development and examine whether collaborative gains translate into independent critical-thinking ability. Some evidence suggests that excessive reliance on peers may hinder autonomous problem-solving, underscoring the need for a balance between teamwork and individual accountability.

Collaborative learning is particularly valuable in anatomy because it requires interpreting complex structures. Studies from Nigeria and elsewhere emphasise that interactive, peer-driven methods foster not only anatomical knowledge but also problem-solving and decision-making skills [11, 24]. However, the extent to which peer collaboration translates into independent critical thinking remains contested. Ensuring that TBL promotes both teamwork and individual mastery is therefore essential.

Students in the TBL group reported higher motivation, reduced stress, and better emotional adjustment to cadaveric dissection. These findings resonate with research showing that reflective, interactive dissection experiences strengthen professional identity, empathy, and resilience [25, 26]. Cadaveric dissection often provokes emotional stress in early-year medical students [12, 4], and TBL may mitigate this by distributing responsibilities and providing peer support. However, team-based emotional buffering depends on group cohesion; poorly functioning teams may inadvertently heighten stress or exclusion. Structured facilitation and clear expectations are therefore essential.

Not all studies have reported positive effects of TBL in anatomy education. For example, study found no significant difference in examination scores between TBL and traditional methods in a medical gross anatomy course, suggesting that the effectiveness of TBL may depend on contextual factors such as class size, facilitator training, and student preparedness [27]. Similarly, study reported that while TBL improved teamwork skills, it did not significantly enhance content mastery compared to problem-based learning [28]. These conflicting findings highlight the need for context-specific implementation and evaluation of TBL, rather than wholesale adoption.

Cadaver scarcity and infrastructural challenges remain persistent issues in Nigerian anatomy education [29, 11, 7]. While students in both groups recognised these barriers, TBL maximised resource utilisation through task-sharing and coordinated engagement with cadaveric material. This supports earlier findings that TBL can adapt effectively to resource-limited environments [30, 19]. Nonetheless, the scalability of TBL in settings with extreme cadaver shortages requires further investigation, as inadequate resources may still compromise the depth of anatomical exploration.

Despite the benefits of TBL, students emphasised that adequate laboratory infrastructure—lighting, ventilation, space, and dissection tools—remains essential for effective learning. This aligns with studies showing that infrastructural deficits can undermine even the most innovative pedagogical strategies [3, 30]. Pedagogical reforms must therefore be accompanied by investments in physical resources to ensure that TBL can be implemented effectively and sustainably.

While the results in Tables 2 and 3, and 4 show consistently higher scores for the TBL group across multiple outcomes, it is not possible to attribute these differences solely to the “team-based” element of TBL. The TL group in this study represented a traditional, teacher-directed, passive learning environment, whereas the TBL group received multiple structured interventions simultaneously: (1) small-group work; (2) peer discussion and accountability; (3) individual and group readiness tasks; (4) peer evaluation; and (5) a low-stakes, collaborative environment without constant instructor oversight. Any or all of these components could be responsible for the observed differences.

Implementing TBL in resource-limited Nigerian anatomy laboratories presents several challenges. First, TBL requires substantial preparation time for facilitators to develop readiness assurance tests and application exercises. In this study, each 3-hour TBL session required approximately 6 h of facilitator preparation time. Second, TBL assumes consistent access to preparatory materials (textbooks, handouts), which may not be available to all students in low-resource settings. Third, TBL requires stable small-group composition over multiple sessions; absenteeism (observed in 12% of TBL sessions in this study) disrupts team function. Fourth, peer evaluation systems may be culturally unfamiliar in Nigerian educational contexts, where hierarchical teacher-student relationships predominate.

Regarding scalability, TBL may be more resource-efficient than traditional dissection in terms of cadaver utilization (as observed in Table 4), but it requires investment in facilitator training (estimated at 8–12 h per instructor) and development of TBL-specific materials. Institutions considering TBL adoption should pilot the method in a single course before scaling to the full curriculum.

### The following limitations of this study are acknowledged

Peer evaluation was conducted only in the TBL group, which may have contributed to differences in reported collaboration and teamwork skills. The absence of this component in the TL group means that observed differences cannot be attributed solely to the team-based instructional method.

As noted above, the TBL group received multiple structured interventions simultaneously, preventing isolation of individual components.

The TL group did not receive an equivalent individual readiness task, which may have influenced baseline knowledge and motivation.

Perceptions of skills development, motivation, and emotional adjustment were measured via self-report questionnaires, which are subject to social desirability and recall biases.

Lack of randomisation and baseline comparability, as well as limited instrument validation.

## Conclusion

This study suggests that a team-based approach to cadaveric dissection is associated with higher examination scores and more positive student perceptions of collaboration, motivation, and emotional adjustment compared to traditional passive dissection methods in a Nigerian university setting. Additionally, TBL appeared to optimise the utilisation of cadaveric resources, which may be particularly relevant for resource-limited institutions. However, due to the quasi-experimental design and lack of random allocation, causal claims cannot be made. Future randomised controlled trials with active control groups are needed to establish causality and isolate the specific components of TBL that drive observed differences. The findings are consistent with global and Nigerian studies that support a shift from traditional teacher-centred approaches toward more interactive and collaborative pedagogies in anatomy education.

## Supplementary Information

Supplementary Material 1.

## Data Availability

The datasets generated and analysed during the current study are available from the corresponding author on reasonable request. The questionnaire used in the study is provided as Supplementary File 1.

## Appendix

The table presents the baseline demographic characteristics of both groups. No statistically significant differences were observed between groups in terms of age distribution (χ² = 1.42, *p* = 0.70), sex ratio (χ² = 0.31, *p* = 0.58), or prior anatomy coursework performance (mean previous semester anatomy score: TL = 62.4% ± 8.1%; TBL = 63.1% ± 7.9%; t = 0.54, *p* = 0.59).

**Table 6:** Baseline characteristics of TL and TBL Groups</ins>

Characteristic	TL Group (*n* = 77)	TBL Group (*n* = 78)	*p*-value
Mean age (years ± SD)	22.4 ± 2.1	22.6 ± 2.3	0.70
Sex (Male/Female)	26/51	24/54	0.58
Previous anatomy score (%)	62.4 ± 8.1	63.1 ± 7.9	0.59
Prior dissection experience (%)	100%	100%	1.00
