# Supplementary material for: Evaluating the educational effectiveness of team-based cadaveric dissection in undergraduate anatomy training
Source: BMC Med Educ. 2026 Jun 9;26:1317. doi: 10.1186/s12909-026-09491-2 (PMC13474753; doi:10.1186/s12909-026-09491-2)
Supplement: Supplementary file 1 [file 12909_2026_9491_MOESM1_ESM.docx]

**Questionnaire**

**SECTION A: Demographic Information**

Please tick (✓) the appropriate option.

**1. Age:** ☐ 18–22 ☐ 23–27 ☐ 28–32 ☐ 33–37 ☐ 38–45

**2. Gender:** ☐ Male ☐ Female

**3. Department:** ☐ Medicine ☐ Nursing ☐ Anatomy ☐ Radiography ☐ Others (specify): ____________

**4. Academic Level:** ☐ 200 Level ☐ 300 Level ☐ 400 Level

**5. Have you participated in cadaveric dissection classes before?** ☐ Yes ☐ No

**6. Mode of dissection used in your course:** ☐ Traditional dissection ☐ Team-based dissection ☐ Both

**SECTION B: Impact of Team-Based Cadaveric Dissection**

Please indicate the option that best represents your view. **A = Agree B = Disagree C = Neither Agree Nor Disagree**

| **Item** | **Description** | **Response** |
| --- | --- | --- |
| **1** | The team-based dissection method improved my understanding of anatomical concepts. | ☐ A ☐ B ☐ C |
| **2** | My learning outcomes were better under the team-based method than under the traditional method. | ☐ A ☐ B ☐ C |
| **3** | The team-based approach enhanced my collaboration and teamwork abilities. | ☐ A ☐ B ☐ C |
| **4** | The group setting during dissection improved my critical thinking and problem-solving skills. | ☐ A ☐ B ☐ C |
| **5** | I found the team-based sessions more emotionally engaging than traditional ones. | ☐ A ☐ B ☐ C |
| **6** | The team-based approach increased my motivation to learn anatomy. | ☐ A ☐ B ☐ C |
| **7** | I felt more connected and supported when learning in a team setting. | ☐ A ☐ B ☐ C |
| **8** | Limited cadaveric resources negatively affected the team-based method's effectiveness. | ☐ A ☐ B ☐ C |
| **9** | Cadaver availability was adequate for effective learning during a team-based dissection session. | ☐ A ☐ B ☐ C |
| **10** | Poor laboratory infrastructure hindered the effectiveness of the team-based dissection approach. | ☐ A ☐ B ☐ C |
| **11** | The existing laboratory infrastructure effectively supported team-based dissection. | ☐ A ☐ B ☐ C |
